# Circumstances and Consequences of Violence-Related Injuries Presenting at Hospital. A Study at the Pediatric Emergency and Forensic Medicine Units of Maputo Central Hospital, Mozambique

**DOI:** 10.3390/ijerph182212125

**Published:** 2021-11-18

**Authors:** Sérgio Keita Nhassengo, Stela Ocuane Matsinhe, Eunice Jethá, Lucie Laflamme

**Affiliations:** 1 Department of Global Public Health, Karolinska Institutet, 171 77 Stockholm, Sweden; lucie.laflamme@ki.se; 2 Faculty of Medicine, Eduardo Mondlane University, Maputo 257, Mozambique; stelasau@yahoo.com.br (S.O.M.); ejetha@yahoo.com.br (E.J.); 3 Institute for Social and Health Sciences, University of South Africa, Cape Town 7500, South Africa; 4 Maputo Central Hospital, Forensic Medicine Service, Maputo 264, Mozambique; 5 Institute for Social and Health Sciences, University of South Africa, Pretoria 0003, South Africa

**Keywords:** child abuse and neglect, violence-related injury, sexual violence, physical violence

## Abstract

Rates of violence against children are high in Sub-Saharan Africa and information is scarce on the resulting injuries. This study investigates sex-related differences in the circumstances and consequences of sexual and physical violence in the Mozambican context. Hospital records from 2019 at the pediatric emergency and forensic medicine units of Maputo Central Hospital were scrutinized using a standardized form. Of the 321 cases identified, 60% resulted from sexual violence. Girls represented 86.4% of the victims of sexual violence and boys, 66.1% of those from physical violence. Being injured in a familiar environment and by a parent, a relative, or someone known was strikingly common. The injury pattern varied by form of violence and sex of the child. About half of the injuries sustained by physical violence were minor/superficial. Severe injuries requiring hospitalization (33% in total) and some specialized care (27% in total) were mainly sustained by girl victims of sexual violence. While circumstances and consequences of violence-related injuries have several similarities, being severely injured is more typical of girl victims of sexual violence. Besides medical care, hospital services in Mozambique must be prepared to offer pediatric victims of violence the necessary social care.

## 1. Introduction

Worldwide, violence-related injury is an increasing problem during childhood and becomes more common as the child gets older, especially in low-income countries [1]. Globally, the one-year prevalence of physical, sexual, or psychological violence or neglect is estimated at up to 1 billion among children aged 2–17, roughly half the children in the world [2]. The number of children affected and the preventability of the numerous individual and societal consequences makes violence against children an acknowledged public health issue [3]. Rates of violence vary considerably between regions, with the highest rates found in countries in Asia (64%), North America (56%), and Africa (50%) [4]. In fact, the lifetime prevalence of child physical violence in Sub-Saharan Africa (SSA) ranges between 42% and 66% in girls and 53% and 76% in boys and that of sexual violence, from 5% to 36%, for both girls and boys [5,6]. In Mozambique, the context of this study, it is estimated that not only have 24% of girls and 34% of boys experienced physical violence but also 14% of girls and 8% of boys have experienced sexual violence in their childhood [7].

The literature indicates that, in general terms, violence-related injuries may occur in different environments. Yet, children sustain sexual and physical violence in their home or at that of the perpetrator, which often is a person they know [8,9]. The vast majority of perpetrators of sexual violence are males, typically a parent or a relative, while physical violence is also inflicted by women (usually a mother) but in lower proportions than men [10,11,12]. This is not surprising given that these children are more likely to be left alone with relatives, neighbors, and friends while their parents work, placing them at a higher risk of assault [13,14,15,16].

In a public health framework, the multi-faceted causes of violence against children are classified as individual, relational, community, and societal level [17,18]. Children exposed to violence can sustain both short- and long-term consequences, such as physical injuries [19,20], which are the focus of this study. In the SSA region, a vast majority of children are subjected to some form of psychological aggression and physical punishment at home on a yearly basis [6]. There are quite a few peer-reviewed scientific studies on violence and violence-related injuries in children in the region, in particular from South Africa [15,16,21,22,23,24,25,26,27,28,29,30] but also from Nigeria [10,31,32,33], Mozambique [11,12,34,35,36,37,38,39], Ethiopia [40,41,42,43], Zimbabwe [5,14,44,45,46], Uganda [35,47], Ghana [13,34], Malawi [48,49], Tanzania [50,51], and Zambia [52]. While data on violence-related injuries are from hospital records [10,15,16,26,29,30,32,33,40,49] or some mortality data [28,48], studies on violence also use household [11,13,14,44,52] and school surveys [21,34,41,50].

The hospital-based studies on injuries highlight the expected association between type of violence and sex of the child, with girls being predominantly victims of sexual violence compared to other forms [8,32,40,41,46] and boys being predominantly victims of physical violence [18,22,31,34]. As under-reporting can occur and can also vary by type of violence, the numbers and rates presented, and the relative distribution between means of violence, this can make the comparisons within and between studies difficult [42,43].

The injuries resulting from violence against children can occur in isolation or in combination of both, physical and sexual [47]. Hospital-based studies on sexual violence against girls show a tendency for younger girl victims of violence to sustain more severe injuries [15], which may be attributable to a less developed genital area, related to low levels of estrogen, the hormone responsible for development and maintain the reproductive system, and elasticity of tissues. Overall, girls presented with genital injuries, such as lacerations, bruises and erythema, and boys presented with anal injuries [10]. Not surprisingly, the absence of injuries in the genital area, particularly in girls, is often reported due to a delay in disclosure [29,30]. For those presenting with acute and severe injuries, a small number needed emergency surgical treatment [10,32]. As for physical violence, while boys are more afflicted than girls, the injuries sustained are similar: mainly injuries to soft tissues, followed by bone fractures and penetrative wounds [2,40,53]. Some have several injuries [29]. There are reports of a low mortality rate, in most of cases, the victims are treated and discharged from hospital [28,35].

In Mozambique, violence against children is widespread, especially children participating in sexual activities with adults in exchange for economic goods or social status [12,39]. Financial compensation or compulsory marriage are also used to silence crimes of sexual violence [11]. To address this problem, Mozambique has created legislation and policies for the promotion and protection of children’s rights. However, those efforts do not translate into significant sustainable actions with meaningful impacts [54,55]. Knowledge of the epidemiology of violence-related injuries among children is scarce and this lack of knowledge makes it difficult to obtain a realistic picture of the burden of the problem [37,38].

This study was performed to address this knowledge gap in the Mozambican context, building on data gathered at two hospital-based sources: pediatric emergency and forensic services. We investigated differences between boys and girls regarding the circumstances and consequences of pediatric injuries sustained due to sexual and physical violence. The following research questions are addressed: (I) What are the forms of violence injured children are exposed to? (II) What are the circumstances of violence-related injuries and how do they differ between boys and girls? (III) What are the characteristics of violence-related injuries and how do they differ between boys and girls? We aim to increase the knowledge of the characteristics and circumstances of violence-related injuries treated in a hospital setting in Maputo.

## 2. Materials and Methods

### 2.1. Design

This study was cross-sectional and retrospective and used hospital records.

### 2.2. Setting

Maputo City, the capital and the largest city, is in southern Mozambique. It has an area of 346.77 square kilometers with a population of 1,088,449, of which 464,035 are children aged 0–18 years old (41%). It is also the main financial, corporate, and commercial center in the country. It is divided into seven municipal districts (KaMpfumo, Nlhamankulu, KaMaxaquene, KaMavota, KaMubukwana, KaTembe, and KaNyaka) which are, in turn, divided into neighborhoods and villages [56].

Like in other provinces of the country, many children in Maputo city are homeless and without civil registration [57]. Child labor is frequent (an estimate of about 276,440 children aged 7–17 years) and so is children being out of school (38.6% at country level, with comparable proportions among girls and boys, 38.8% and 38.4%, respectively) [58]. In Maputo, over 10,000 children aged 12–14 years are the head of a household and a majority of these are girls [59].

MCH, where this study was conducted, is a health unit of national reference and it is the largest one in Mozambique. It is in the center of Maputo city. It is a quaternary level hospital, with the status of a university hospital, providing diagnostic, curative, palliative, rehabilitation, and preventive care [60]. MCH has 7 clinical departments with 42 inpatient services, of which the Department of Medicines has 11 inpatient services (including forensic medicine), and that of pediatric has 9 services (including pediatric emergency). For this study, data were collected at the services (or units) of forensic medicine and pediatric emergency, the two where children injured by acts of violence are most likely to be seen.

Pediatric emergency has 307 beds, with a total of 39 pediatric doctors and 148 nurses and forensic medicine, 10 forensic doctors. It is where injured children are expected to be seen for an injury requiring medical care [54]. Forensic medicine is where children are seen when there is suspicion of abuse or neglect, notably from the police, which is relatively infrequent. Additionally, some of the children referred to the forensic unit come directly from the emergency unit [61].

### 2.3. Case Definition and Data Collection

In this study, we adopted the definition of WHO violence-related injury that is defined as an injury sustained by an act committed deliberately by another person (or a group of persons) [17]. We included as a case any child aged 0–14 years seen at one of the units because of an injury (or several injuries) sustained by an act of inter-personal violence during the 12-month period of 1 January 2019 to 31 December 2019. Cases seen for the same injury(ies) at the two units were counted only once, at the forensic. For the forensic unit, we also included children examined because of suspected acts of (sexual) abuse or violence even though no apparent physical injuries were noted. Re-admission (or visits) within 30 days for the same injury(ies) were excluded and so were cases of self-inflicted injuries.

For the data collection, we requested authorization from the head of each of the two units to access the bulletins and record books. Typically, cases were sought at each service by reviewing the logbook where all entries to the service are listed and given sequential record numbers. All identified cases were then sought and retrieved from the storeroom or archive. At the pediatric unit, there is no systematic archiving of the records which are kept in boxes (some of which are not organized by years). The records of some cases identified in the logbook could not be found (approximately 30 cases). At forensic medicine, the cases are registered chronologically in numerical order and all cases could be found; 44 cases were transfers from pediatric emergency and those cases were counted only once, at the forensic unit.

We used a standardized Case Report Form, developed in accordance with the WHO guideline for injury surveillance [62]. It included information about the form of violence that the child had been exposed to (sexual and physical); the child’s details (age, sex, link with the perpetrator, accompanying person); the injury sustained (number of injuries, body part injured, nature of injury, severity, and disposition) and circumstances (place of occurrence, time of day, day of week, means/strategy used by the perpetrator to attract the child).

### 2.4. Data Treatment

All analyses were conducted stratified by the form of violence: sexual or physical. After looking at the distribution of the victims by age and sex, we used bivariate analyses to describe the sex-specific circumstances of the injuries as indicated in the case descriptions available in the records. We considered in turn, who the perpetrator was, the number of perpetrators, day of the week, time of day, place of occurrence, means used by the perpetrator to attract the child. Similarly, we had a closer look at the sex-specific consequences of the injuries, considering the part of the body injured (number and specific location), nature of the injury, severity and disposition. We coded separately whether the following part of the body was injured—yes or no (genital, head, thorax and abdomen low limbs, upper limbs, and anal). We proceeded in the same manner for the nature of the injury and coded whether the following was reported (bruises or superficial injury, hymnal rupture, vaginal vulvar laceration, genital hematoma, anal fissure, vulvar edema, fractures, burns, hyperemia, and perineal rupture). The data were treated using a Microsoft Excel^®^ (version number 2016).

### 2.5. Statistical Analyses

The association between the sex of the child and any of the attributes considered was assessed by means of chi-square tests for comparison of proportions. However, we did not test for significance in sex-related differences for sexual violence as the number of boy victims was relatively low (*n* < 30). For the part of the body injured and the nature of the injury, we dichotomized each category to test for differences. We did not test for categories that were not present in both sexes. We used an online chi-square test calculation tool and retained a significance level of 0.05 [63].

### 2.6. Ethical Approval

The study received approval from the Comité Institucional de Bioética em Saúde da Faculdade de Medicina/Hospital Central de Maputo-CIBS FM&HCM (Institutional Health Bioethics Committee of the Faculty of Medicine/Maputo Central Hospital). In a meeting held on 3 September 2020, protocol was registered with number CIBS FM&HCM/053/2020.

## 3. Results

We identified a total of 321 cases of violence-related injuries affecting children at MCH, of which 143 were from pediatric emergency (44.5%) and 178 at forensic medicine (55.5%). Of these, 191 (59.5%) were related to sexual violence and 130 (40.5%), physical violence (Table 1). The victims were brought to the hospital mainly by their parents and victims presented at forensic medicine were reported to the police beforehand.

### 3.1. Age and Gender Distribution by Forms of Violence

**Figure 1:**
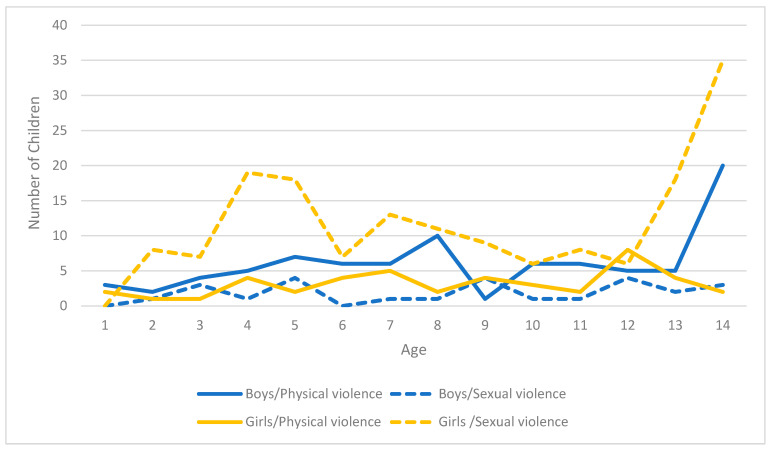
Distribution of the victims of violence-related injuries by age and sex of the child and form of violence, data from pediatric emergency and forensic medicine at MCH, from 1 January to 31 December 2019.

All ages aggregated, there were nearly twice as many girl victims of violence than boys (209/112). Yet, over six girls were victims of sexual violence for one boy (girls to boys ratio 165/26 = 6.35) while almost two boys for one girl sustained physical violence (boys to girls ratio 86/44 = 1.95). There were victims of violence at all ages but for children aged 1 year or less, more girls were injured than boys. Figure 1 shows the distribution of the cases by child age and sex, stratified by type of violence. The higher numbers among girls are more striking from 10 years of age and largely attributable to the higher level of sexual violence consistent across ages. For their part, boys were more often injured by physical violence than girls (with the exceptions of ages 9 and 12), more strikingly so at age 14.

### 3.2. Circumstances of Occurrence of the Injuries

Table 2 presents the circumstances surrounding the injuries by type of violence and sex of the child. While the identity of the perpetrator varied according to type of violence and sex of the child, the table shows that, in most instances, this person was someone known by the child, very often a relative. It is only for acts of sexual violence among girls that the perpetrator was reported as unknown in many instances (22.4%). There is most often only one perpetrator except for acts of sexual violence, particularly among girls (9%). In addition, for both forms of violence and both boys and girls, the violence was most often perpetrated in the victim’s house. Sexual violence also occurred often in the perpetrator’s house, and physical violence, on the street.

While the total number of victims was higher on weekdays than on weekends, girls were more often victims of sexual violence on weekends than boys and less often victims of physical violence. Likewise, in the evening, the total number (and proportion) of girls who were victims of sexual violence was much greater than that of boys, but that of boys who were victims of physical violence, greater than that of girls.

### 3.3. Characteristics of the Injuries and Injury Treatment

Table 3 presents the characteristics of injuries, stratified according to the sex of the victim and type of violence. As can be seen, children sustaining two injuries is common for both types of violence and among both boys and girls. There is no significant difference in that respect between boy and girl victims of physical violence (58.1% compared to 52.3% respectively). Several girl victims of sexual violence also sustained two injuries (67.9%). Whereas the girls’ genitals are by far the most frequent part of body injuries by sexual violence, boys and girls sustained injuries from physical violence to several body parts, the head and upper limbs being frequent sites for both boys and girls. No significant differences are observed in that respect.

From the hospital records, bruises were by far the most common type of injury registered in all instances. Girl victims of sexual violence also sustained a range of injuries to the genitals and anus, as did the boys in the latter case.

Most injuries due to physical violence were classified as minor or superficial (57.1% and 59.0% for girls and boys, respectively) and there were far more injuries classified as moderate and requiring specialized or severe care and requiring intensive care (18 of 26 among boys and 96 of 165 among girls). Medical consultation with immediate discharge was most frequent for both types of violence among boys and girls. In contrast, girls who suffered sexual violence received proportionally more surgery (*n* = 27 compared with *n* = 2 of boys).

## 4. Discussion

### 4.1. Main Findings

One first finding of this study is the predominance of sexual violence over physical violence afflicting the children who are victims of inter-personal violence presenting at the two units of the hospital. As indicated in Section 2, given that victims of sexual violence were overwhelmingly girls and the number of boy victims was low (*n* = 26), we did not test for sex-related differences in the circumstances and consequences of sexual violence, but we reported on the distributions for both sexes. While both forms of violence were often perpetrated by relatives and persons children know, unknown perpetrators were most frequent among children injured by sexual violence: 43 in total, of which 37 among girls, compared to 9 for physical violence (7 boys and 2 girls, with significant differences in percentages) The child’s home was the most frequent place of occurrence of the violence for all victims except for the boy victims of sexual violence. Girls were mainly harmed during weekdays by both sexual and physical violence (the latter with a significant difference compared to boys). While there was no significant difference between boys and girls in the time of occurrence of physical violence more than half of the sexual violence against girls occurred in the evening.

About half of the children sustained two or more injuries and multiple injuries were more common among victims of sexual violence. Additionally, the parts of body injured, and nature of injuries varied remarkably when comparing the type of violence and, for the victims of sexual violence, the sex of the victim. While, medical consultation with immediate discharge was relatively common for both forms of violence and for boys and girls. Yet, nearly all severe injuries requiring intensive care and those with surgery were sustained by girl victims of sexual violence (27 out of 29 and 29 of 31).

### 4.2. Comparisons with Other Studies

Finding an association between form of violence, age, and sex of the victim was expected, as in other parts of the region (e.g., South Africa [22,24], Ethiopia [40,41], and Kenya [34]) and so was the over-representation of girls among the victims of sexual violence and the increase in violence as they got older [42,43]. This is a reflection that child sexual violence can be understood as gender violence, in line with the historical inequality between men and women, one of the consequences being the abuse of young girls from an early age [6,11,12,27,40,45]. These observations go beyond the SSA region and were also documented in resource-poor countries from other regions like Egypt [64], India [65], and Pakistan [66]. It was also expected that the perpetrators of both forms of violence were often known by the child [11,12,13,14] or predominantly a man (especially for sexual violence) [15,16]. In fact, as indicated earlier, acts of violence occur usually in settings that the child is familiar with. Additionally, girls spend more time in the home [8,9,10].

That children may suffer several injuries is echoed in earlier studies from the region [10,33,67]. The parts of the body injured, and nature of injuries, varied remarkably when comparing the type of violence and, for the victims of sexual violence, the sex of the victim. For example, bruises were the principal injury described among girls for both physical and sexual violence, which is quite similar in some parts, but contradicts some similar studies that revealed perineal injuries and lacerations as the main sexual-related injury [2,15,40,55,65,68].

While variations in injury severity and care were to be expected, it is of note that nearly all severe injuries requiring intensive care or surgery were sustained by girl victims of sexual violence [30,32].

### 4.3. Strengths and Limitations

This study is one of the few on pediatric violence-related injuries conducted in a hospital setting, not only in Mozambique but also in the SSA region. Based on data from two potential entry points of injured children, the emergency and forensic units, it offers a more detailed picture of the physical harm they suffer and the care they receive. The secondary data used were of relatively good quality, with minimal missing information. As indicated above, Maputo Central Hospital is the referral hospital of the city and it is therefore where severely injured children will be referred. The data are therefore silent about the burden of less severe injuries not referred to hospital.

Nonetheless, as is often the case for studies on violence, we most likely provide an underestimation of the magnitude of the problem. Critical issues rely on the fact that not all children injured by acts of violence will be seen and receive care at the central hospital and that not all intentional injuries will be described as such, be it by the victim or the person accompanying them or by the hospital staff. Reasons for under-reporting are indeed numerous [17].

Additional methodological limitations lie the cross-sectional design, which limits our ability to tell whether the portrait presented is consistent over time and the unique site of the study that makes the representativity of the results at regional or country-level uncertain. We expect that the circumstances and consequences of violence-related injuries exposed are valid in other settings, but the proportions presented, and the sex-related differences could vary. For the cases not found in the archives, we do not expect any systematic bias in that respect (e.g., based on service, clinician, or injury type) and we have good reason to believe that the variables selected will be relatively well documented (internal validity). Finally, it is of note that none of the children injured lacked a registration number or place of residence. We do not believe that this can be interpreted as children without a registration number or place of residence not being injured by acts of violence, physical or sexual. On the contrary, we believe that, due to their living circumstances, those children are very vulnerable to abuses of this kind, but that barriers to their coming or being transported to the hospital are numerous.

### 4.4. Implications of Research, Policy, and Practice

The UNICEF Violence Gap Map highlights that building the evidence base on interventions and outcomes for protecting children from violence and exploitation to inform policy and practice must receive priority [69]. While primary prevention is undoubtedly critical for the long-term reduction of violence, the process of changing deep-rooted societal norms takes time, and secondary and tertiary prevention measures are required. These include for example support and care services for child victims to minimize consequences of the injuries and prevent reoccurrence [70,71]. Data are needed to inform the choice and contextualization of secondary and tertiary prevention measures.

The results of this study make a useful contribution. Hopefully, it can also give an impetus to a more holistic approach to their care, one that goes beyond the short-term and “visible” consequences of violence. Together with the findings from other countries from the region, the injury data accumulated may help reinforce evidence-based actions, at local and regional levels, considering the recommendations for evaluating the performance and for the quality of services provided to victims of violence [72,73,74].

## 5. Conclusions

The study shows an expected association between sex of the child and type of violence even in the Mozambican context. Victims are often hurt by a close or known person in a familiar environment. Injuries classified as severe or requiring hospitalization and specialized care are less common and typically sustained by girl victims of sexual violence. Besides medical care, hospital services in Mozambique must be prepared to offer pediatric victims of violence the necessary social care. Protecting children from violence- and abuse-related injuries will require not only a change in norms but also the promotion of children’s rights.

## Figures and Tables

**Table 1:** Type of violence among the pediatric patients seen at pediatric emergency and forensic medicine at MCH from 1 January to 31 December 2019.

Type of Violence	Pediatric Emergency *n*. = 143		Forensic Medicine *n*. = 178		Total *n*. = 321	
	*n*.	%	*n*.	%	*n*.	%
Sexual	79	55.2	112	62.9	191	59.5
Physical	64	44.8	66	37.1	130	40.5
Total	143	100.0	178	100.0	321	100.0

**Table 2:** Sex-related difference in the circumstances of violence-related injuries among pediatric patients seen at pediatric emergency and forensic medicine at MCH, from 1 January to 31 December 2019.

Circumstances of Occurrence	Sexual Violence *n*. = 191			Physical Violence *n*. = 130				
	Boys	Girls		Boys		Girls		*p*-Value
	*n*. = 26	*n*. = 165	%	*n*. = 86	%	*n*. = 44	%	
Perpetrator								
Father	0	25	15.1	23	26.7	9	20.4	n.s. ^1^
Mother	0	0	0.0	14	16.3	5	11.5	-
Other relative	7	50	30.4	16	18.6	14	31.8	0.0922
Person known	12	48	29.1	24	27.9	14	31.8	n.s.
Person unknown	6	37	22.4	7	8.1	2	4.5	0.0004
Do not know/missing	1	5	3.0	2	2.3	0	0.0	-
Number of perpetrator(s)								
1	24	150	90.9	86	100.0	44	100.0	n.s.
2 or more	2	15	9.1	0	0.0	0	0.0	-
Place of occurrence								
Victim’s house	8	70	42.4	52	60.5	27	61.4	n.s.
Perpetrator’s house	9	50	30.3	7	8.1	3	6.8	n.s.
Street	2	20	12.2	18	20.9	6	13.6	n.s.
Other (e.g., public or religious environment)	6	22	13.3	7	8.1	8	18.2	0.0001
Do not know/missing	1	3	1.8	2	2.4	0	0.0	-
Day of week								
Weekday	18	106	64.2	56	65.1	38	86.4	0.0115
Weekend	6	48	29.1	30	34.9	6	13.6	0.0115
Do not know/missing	2	11	6.7	0	0.0	0	0.0	-
Time of day								
Morning	0	20	12.1	30	34.9	17	38.6	n.s.
Afternoon	12	53	32.2	24	27.9	17	38.6	n.s.
Evening	13	85	51.5	29	33.7	10	22.7	n.s.
Do not know/missing	1	7	4.2	3	3.5	0	0.0	-

^1.^ not significant.

**Table 3:** Sex-related difference in the characteristics of violence-related injuries among pediatric patients seen at pediatric emergency and forensic medicine at MCH, from 1 January to 31 December 2019.

Characteristics of Injuries	Sexual Violence *n*. = 191			Physical Violence *n*. = 130				
	Boys	Girls		Boys		Girls		*p*-Value
	*n*. = 26	*n*. = 165	%	*n*. = 86	%	*n*. = 44	%	
Number of injuries								
1	12	25	15.1	36	41.9	21	47.7	n.s. ^1^
2	13	112	67.9	50	58.1	23	52.3	n.s.
3 or more	1	28	17.0	0	0.0	0	0.0	-
Nature of injury (yes)								
Bruises (yes)	15	114	69.1	73	84.9	34	77.3	n.s.
Hymnal rupture (yes)	0	43	26.1	0	0.0	0	0.0	-
Vaginal vulvar laceration (yes)	0	40	24.2	0	0.0	0	0.0	-
Genital hematoma (yes)	0	37	22.4	0	0.0	0	0.0	-
Anal fissure (yes)	26	13	7.9	0	0.0	0	0.0	-
Vulvar edemas (yes)	0	26	15.8	0	0.0	0	0.0	-
Fractures (yes)	0	0	0.0	5	5.8	3	6.8	-
Burns (yes)	0	0	0.0	5	5.8	4	9.1	-
Hyperemia (yes)	0	0	0.0	3	3.5	3	6.8	-
Perineal rupture	0	3	1.8	0	0.0	0	0.0	-
Part of body injured								
Genitals (yes)	0	137	83.0	0	0.0	0	0.0	-
Head (yes)	2	5	3.0	45	52.3	24	54.5	n.s.
Upper limbs (yes)	2	6	3.7	28	32.6	19	43.2	n.s.
Thorax and abdomen(yes)	1	3	1.8	24	28.0	9	20.5	n.s.
Lower limbs (yes)	1	0	0.0	15	17.4	5	11.4	n.s.
Anal (yes)	26	14	8.5	0	0.0	0	0.0	-
Severity								
Minor or superficial	8	69	41.8	49	57.0	26	59.1	n.s.
Moderate, requiring specialized care	16	67	40.6	35	40.7	18	40.9	n.s.
Severe, requiring intensive care	2	29	17.6	2	2.3	0	0.0	-
Disposition								
Medical consultation with immediate discharge (yes)	29	127	78.0	41	41.9	32	68.2	0.005
Psychological support (yes)	17	76	46.0	38	44.2	20	45.5	n.s.
Surgery(yes)	2	27	16.3	0	0.0	0	0.0	-
Hospitalization (yes)	7	33	20.0	12	14.0	12	27.3	n.s

^1.^ not significant.
